# Integration of Hematologic and Metabolic Biomarkers for Outcome Prediction in Acute Coronary Syndromes Without ST Elevation

**DOI:** 10.7759/cureus.86446

**Published:** 2025-06-20

**Authors:** Emir Becirovic, Minela Becirovic, Kenana Ljuca, Amir Becirovic, Mirza Babic, Nadina Ljuca, Zarina Babic Jusic, Emir Begagic, Elma Mujakovic, Anesa Terzic

**Affiliations:** 1 Internal Medicine Clinic, University Clinical Centre Tuzla, Tuzla, BIH; 2 Intensive Care Unit, University Clinical Centre Tuzla, Tuzla, BIH; 3 Nephrology, University Clinical Centre Tuzla, Tuzla, BIH; 4 Gynecology and Obstetrics, University Clinical Centre Ljubljana, Ljubljana, SVN; 5 Endocrinology, University Clinical Centre Tuzla, Tuzla, BIH; 6 Internal Medicine, Cantonal Hospital Bihać, Bihać, BIH; 7 Medicine, University of Tuzla, Tuzla, BIH; 8 Radiology, Cantonal Hospital Bihać, Bihać, BIH; 9 General Medicine, School of Medicine, University of Zenica, Zenica, BIH; 10 Anatomy, Faculty of Medicine, University of Tuzla, Tuzla, BIH; 11 General Medicine, Health Center Gračanica, Gračanica, BIH

**Keywords:** inflammation, mace, nstemi, piv, prognostic biomarkers, siri, tyg-bmi

## Abstract

Background

Non-ST-elevation myocardial infarction (NSTEMI) represents a prevalent form of acute coronary syndrome associated with substantial early risk of adverse outcomes. Inflammatory and metabolic disturbances are increasingly recognized as key contributors to the disease. Hematologic indices such as the neutrophil-to-lymphocyte ratio (NLR), platelet-to-lymphocyte ratio (PLR), lymphocyte-to-monocyte ratio (LMR), systemic immune-inflammation index (SII), systemic inflammation response index (SIRI), and pan-immune-inflammation value (PIV), along with the triglyceride-glucose index adjusted for BMI (TyG-BMI), have emerged as promising prognostic markers. However, their dynamic behavior in early NSTEMI remains insufficiently explored.

Materials and methods

This prospective study included 170 patients hospitalized for NSTEMI at the University Clinical Centre Tuzla between February 2022 and January 2023. Hematologic and metabolic indices were calculated at admission and repeated 24 hours later. Patients were followed for three months to document major adverse cardiovascular events (MACE), including cardiovascular death, reinfarction, and urgent revascularization. The median age was 67 years, and 60.6% of patients were male. Hypertension, hyperlipidemia, and diabetes mellitus were the most common comorbidities.

Results

Significant 24-hour reductions were observed in NLR, PLR, SII, SIRI, and PIV (all p < 0.01), while C-reactive protein (CRP) levels more than doubled (p < 0.001). Patients who developed MACE showed persistently elevated inflammatory indices and smaller declines in PIV and SIRI. Change in SIRI (ΔSIRI) demonstrated the strongest predictive value (AUC = 0.63), followed by SII and TyG-BMI. Notably, reduced resolution of PIV and persistently elevated TyG-BMI were significantly associated with adverse outcomes. Overall, MACE occurred in 51.2% of patients, including a 14.7% mortality rate.

Conclusion

Early changes in systemic inflammation and metabolic stress, particularly SIRI and TyG-BMI dynamics, offer valuable prognostic insight and may enhance early risk stratification in NSTEMI patients.

## Introduction

Non-ST-elevation myocardial infarction (NSTEMI) is a prevalent subtype of acute coronary syndrome (ACS) and represents a significant cause of hospital admissions and cardiovascular mortality [1]. Despite early invasive strategies and optimal pharmacologic interventions, many NSTEMI patients continue to experience recurrent ischemic events and poor long-term outcomes [2]. Identifying additional prognostic markers beyond conventional clinical and angiographic parameters remains a key goal for improving care and guiding individualized management [3].

Accumulating evidence supports the central role of systemic inflammation and metabolic stress in the initiation, progression, and resolution of ACS [4]. Inflammatory activation not only contributes to plaque instability but also influences myocardial healing and remodeling following infarction [5]. Hematologic indices derived from routine complete blood counts, such as the neutrophil-to-lymphocyte ratio (NLR), platelet-to-lymphocyte ratio (PLR), lymphocyte-to-monocyte ratio (LMR), systemic immune-inflammation index (SII), systemic inflammation response index (SIRI), and pan-immune-inflammation value (PIV), have emerged as accessible markers that may reflect the extent of immune activation and predict cardiovascular outcomes [6].

At the same time, metabolic derangements, such as insulin resistance, hypertriglyceridemia, and increased body mass index (BMI), are associated with accelerated atherosclerosis and unfavorable prognosis following myocardial infarction [7]. The triglyceride-glucose index adjusted for BMI (TyG-BMI) has been proposed as a reliable surrogate for metabolic burden and early insulin resistance. Elevated TyG-BMI values have been linked to increased risk of coronary artery disease, in-stent restenosis, and adverse clinical outcomes in patients with ACS [8].

Notably, few studies have simultaneously examined both inflammatory and metabolic biomarkers in NSTEMI, and most prior work relied on single-time-point measurements, small sample sizes, or excluded indices such as TyG-BMI, which reflect insulin resistance. Furthermore, little data exist on the serial evolution of novel indices, such as SIRI or PIV, within the early phase of hospitalization [9]. Whether combining these biomarkers improves short-term risk stratification remains incompletely defined [10]. However, serial assessment of these indices, rather than single time-point measurements, may better reflect the biological course of myocardial injury and the systemic response [11]. The indices included in this study (SIRI, PIV, NLR, PLR, and TyG-BMI) were selected based on their ability to reflect key components of systemic inflammation and metabolic dysfunction. SIRI and PIV are emerging indices that integrate leukocyte subsets and platelets into a single value and have shown prognostic utility in early reports, though they remain underutilized in ACS.

The objective of this prospective observational study conducted at a tertiary care center in Southeastern Europe was to assess the prognostic significance of several hematologic and metabolic indices, including NLR, PLR, LMR, SII, SIRI, PIV, and TyG-BMI, measured both at admission and 24 hours later in patients with NSTEMI [12]. We explored their association with major adverse cardiovascular events (MACE) during a three-month follow-up period. The three-month follow-up was selected to identify important early complications and align with earlier studies that found the highest risk of heart problems during this time after NSTEMI. We hypothesized that persistent inflammatory or metabolic activation within the first 24 hours following NSTEMI would be predictive of poorer outcomes [13]. Based on accumulating clinical evidence, we selected these biomarkers for their ability to reflect key inflammatory and metabolic pathways known to influence cardiovascular risk.

## Materials and methods

This prospective observational study was conducted from February 2022 to the end of January 2023 at the Coronary Care Unit of the Clinic for Internal Medicine, University Clinical Centre Tuzla. The study involved 170 patients who were diagnosed with a new case of NSTEMI based on their symptoms, changes in their ECG, and high levels of cardiac troponin I. Patients with a prior history of myocardial infarction or coronary revascularization were excluded. No minimum duration from symptom onset was required, as all patients were enrolled upon hospital presentation. All eligible patients admitted during the study period were enrolled without post-screening exclusions, minimizing selection bias. The primary objective was to evaluate the prognostic value of several hematologic inflammation-based indices and metabolic markers in predicting MACE in this population.

To be included in the study, patients had to be adults over 18 years old with a confirmed NSTEMI diagnosis based on their symptoms, ECG changes, and high levels of cardiac troponin I. Exclusion criteria were active infection, autoimmune disease, malignant disease, recent trauma or surgery, end-stage liver or kidney disease, and incomplete laboratory data. All patients were treated according to contemporary NSTEMI management guidelines, including optimal pharmacological therapy and invasive coronary assessment when clinically indicated. Treatment decisions, including pharmacologic therapy and revascularization strategy, were guided by current guidelines but ultimately left to clinician discretion based on individual patient factors.

Upon hospital admission, all patients underwent a comprehensive clinical evaluation, including a detailed medical history, physical examination, and anthropometric measurements. Venous blood samples were collected at admission and repeated approximately 24 hours later, within a ±2-hour window from the initial sampling time to ensure operational consistency. Laboratory analyses included complete blood count (CBC), serum glucose, triglycerides, and high-sensitivity C-reactive protein (hs-CRP). BMI was calculated based on measured weight and height. All analyses were performed using certified, hospital-based automated analyzers: Sysmex XN-1000 (Sysmex Corporation, Kobe, Japan) for hematologic parameters and Beckman Coulter DxC 700 AU (Beckman Coulter, Brea, CA, USA) for biochemical parameters.

From the laboratory values obtained at both time points, several inflammation-based indices were calculated, including the NLR, PLR, LMR, SII, PIV, and SIRI. In addition, a combined metabolic marker, TyG-BMI, was calculated using serum glucose and triglyceride levels, along with BMI. All indices were determined at both the admission and 24-hour time points, and changes between the two measurements were recorded.

Patients were prospectively followed for the occurrence of MACE during the three months following discharge. MACE was defined as cardiovascular death, recurrent myocardial infarction, or urgent revascularization. Recurrent myocardial infarction was defined as the development of new ischemic symptoms with a rise in cardiac troponin and corroborating ECG or imaging findings. Urgent revascularization referred to unplanned percutaneous coronary intervention (PCI) or coronary artery bypass graft (CABG) performed within 72 hours due to symptom recurrence or objective evidence of ischemia. Information on event occurrence, time to event, and survival status was obtained from medical records, follow-up clinic visits, and telephone contact. MACE events were adjudicated by experienced cardiologists based on clinical presentation, ECG changes, cardiac biomarkers, and coronary imaging when available. The 24-hour time-point was selected to capture early systemic and inflammatory responses following initial stabilization and to avoid confounding from later in-hospital complications. The study was conducted in accordance with the ethical principles outlined in the Declaration of Helsinki, and the study protocol was approved by the Ethics Committee of the University Clinical Centre Tuzla (approval number: 02-09/2-97/21). Written informed consent was obtained from all participants prior to enrollment.

Statistical analysis

The analyzed variables were tested for normality using the Kolmogorov-Smirnov test. As all continuous variables deviated from a normal distribution, non-parametric statistical methods were applied. Categorical variables were compared using Pearson's χ² test, while continuous variables were analyzed using the Mann-Whitney U test for comparisons between independent groups and the Wilcoxon signed-rank test for paired observations. Categorical variables are presented as absolute numbers (n) and percentages (%), whereas continuous variables are reported as medians with interquartile ranges (IQR).

For inflammation-based and metabolic indices that showed statistically significant differences between patients with and without MACE, receiver operating characteristic (ROC) curve analysis was performed. The area under the curve (AUC), along with 95% confidence intervals (CI), was calculated to assess the discriminative performance of each index. Optimal cutoff values were determined by maximizing the Youden index. A p-value of < 0.05 was considered statistically significant. Cutoff values derived from ROC analysis were exploratory and not internally validated. Multivariable modeling was not performed due to sample size constraints and the risk of model overfitting. All biomarker data were analyzed without transformation. Outliers were retained to preserve clinical variability, and no adjustment for multiple comparisons was applied, as this was an exploratory analysis based on complete-case data.

## Results

A total of 170 patients diagnosed with NSTEMI were included in the final analysis. The median age of the study population was 67 years (IQR: 58-74), with a predominance of male patients (60.6%). Hypertension was the most prevalent comorbidity and was significantly more common among patients who developed MACE compared to those who did not (93.1% vs. 81.9%, p = 0.03). Diabetes mellitus, hyperlipidemia, and elevated BMI were observed at similar frequencies in both groups. Active smoking was more frequent among patients without MACE (57.8% vs. 42.5%, p = 0.046). None of the patients had a history of prior myocardial infarction or coronary revascularization. The baseline demographic and clinical characteristics of the study population are presented in Table 1.

**Table 1:** Baseline characteristics of NSTEMI patients stratified by three-month MACE outcomes. Note: PCI and CABG were not included in the definition of MACE and are presented here as in-hospital clinical interventions. CAD, coronary artery disease; MI, myocardial infarction; PCI, percutaneous coronary intervention; CABG, coronary artery bypass graft surgery; BP, blood pressure.

Characteristic	MACE (n = 87)	No MACE (n = 83)	Test Statistic	p-value
Age, years	66 (59.5–73.0)	70 (60.0–79.0)	U = 3123.0	0.14
Male sex, n (%)	54 (62.1%)	49 (59.0%)	χ² = 0.17	0.68
Hypertension, n (%)	81 (93.1%)	68 (81.9%)	χ² = 5.06	0.03
Diabetes mellitus, n (%)	34 (39.1%)	37 (44.6%)	χ² = 0.52	0.47
Current smoker, n (%)	37 (42.5%)	48 (57.8%)	χ² = 4.00	0.046
Hyperlipidemia, n (%)	61 (70.1%)	63 (75.9%)	χ² = 0.70	0.40
Family history of CAD, n (%)	71 (81.6%)	61 (73.5%)	χ² = 1.65	0.20
BMI, kg/m²	30.4 (27.6–32.1)	29.8 (27.3–32.0)	U = 3394.5	0.67
Systolic BP on admission, mmHg	130 (110–145)	135 (120–150)	U = 3155.0	0.13
Admission glucose, mmol/L	7.8 (6.2–11.9)	7.4 (6.1–10.5)	U = 3346.0	0.35
PCI during index hospitalization, n (%)	36 (41.4%)	6 (7.2%)	χ² = 28.4	<0.001
CABG during index hospitalization, n (%)	13 (14.9%)	2 (2.4%)	χ² = 7.14	0.008

During the index hospitalization, 41.4% of patients in the MACE group underwent PCI compared to 7.2% in the no-MACE group (p < 0.001), while CABG was performed in 14.9% and 2.4% of patients, respectively (p = 0.008). These in-hospital interventions are detailed in Table 1. Overall, 87 patients (51.2%) experienced MACE during the three-month follow-up period, including 25 deaths (14.7%). The median time to MACE was 12 days (IQR: 8-27).

**Figure 1:**
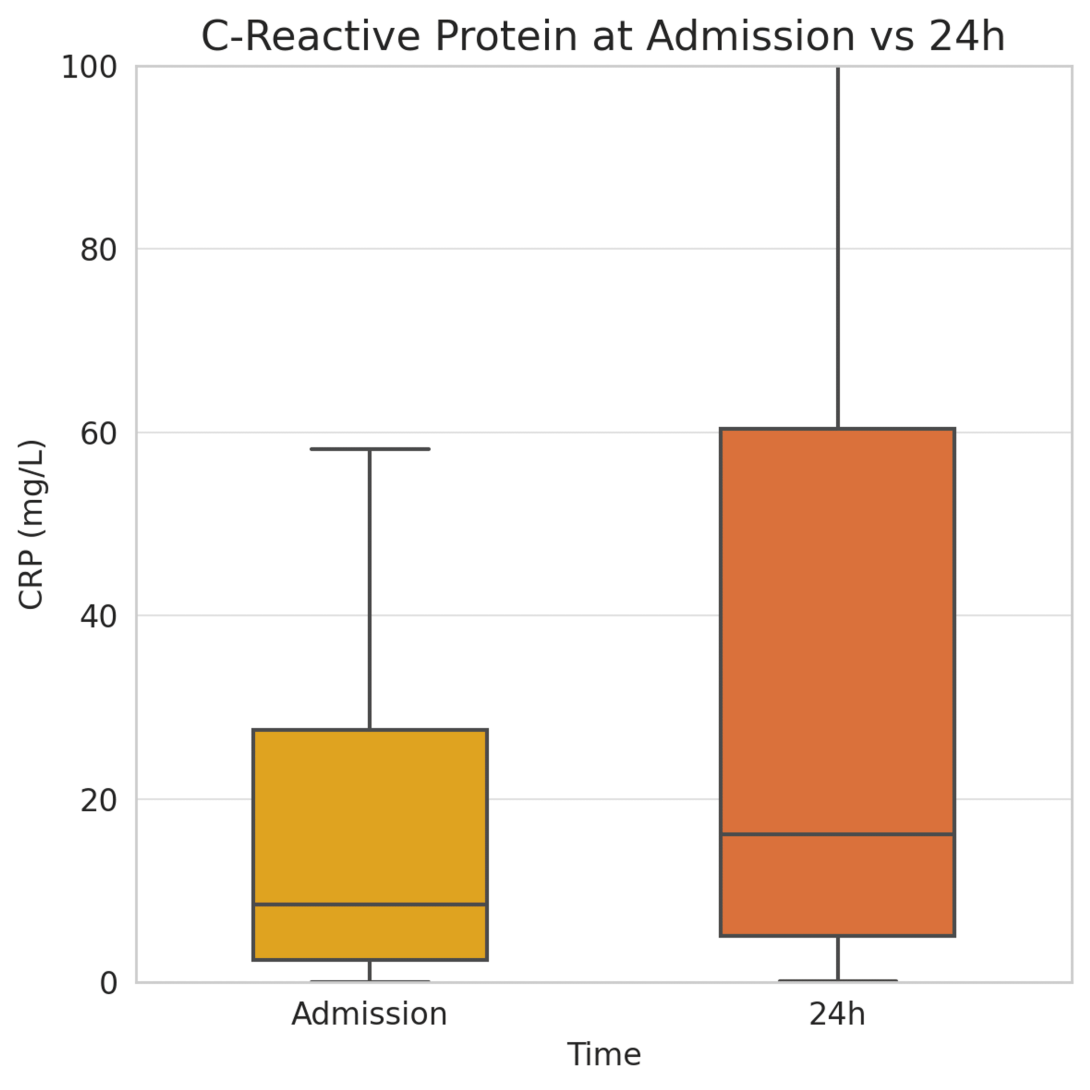
Box plot of CRP levels at admission and after 24 hours. The median CRP more than doubled by 24 hours, and the spread of values widened (IQR ~3–58 mg/L at 24 hours vs. 2.4–27.6 at baseline), consistent with an evolving inflammatory response to myocardial necrosis. CRP, C-reactive protein; IQR, interquartile range.

Conversely, the median CRP concentration more than doubled during the first 24 hours, rising from 8.6 mg/L (IQR: 2.4-27.6) to 16.2 mg/L (IQR: 5.1-60.4) (p < 0.001), consistent with the expected delayed acute-phase response (Figure 1).

The laboratory evaluation of inflammation and metabolic stress revealed dynamic changes between admission and the 24-hour follow-up (Table 2). Most inflammatory indices showed a significant decline. The median NLR decreased from 4.33 to 3.29 (p < 0.001), and the PLR decreased from 143.5 to 130.1 (p = 0.00024). The SII dropped from 995.7 × 10³ to 687.3 × 10³ (p < 0.001), while the SIRI fell from 2.89 to 2.32 (p = 0.0025). A significant reduction was also observed in the PIV, from 704.7 to 546.7 (p = 0.0093). The LMR increased modestly from 2.26 to 2.36, but this change was not statistically significant (p = 0.331). The TyG-BMI index, representing a composite measure of metabolic and anthropometric burden, declined slightly but significantly from 282.5 to 279.7 (p < 0.001), likely reflecting early glycemic management or hemodilution. A summary of all inflammatory and metabolic indices at admission and after 24 hours is provided in Table 2.

**Table 2:** Inflammatory index values at admission vs. after 24 hours. WBC, white blood cell count; RDW, red cell distribution width; MPV, mean platelet volume; CRP, C-reactive protein; NLR, neutrophil-to-lymphocyte ratio; PLR, platelet-to-lymphocyte ratio; LMR, lymphocyte-to-monocyte ratio; SII, systemic immune-inflammation index; SIRI, systemic inflammation response index; PIV, pan-immune-inflammation value; TyG–BMI, triglyceride-glucose index multiplied by body mass index.

Index	Admission Median (IQR)	24-Hour Median (IQR)	Z-statistic	p-value
WBC (×10⁹/L)	9.8 (7.5–12.3)	9.5 (7.3–11.9)	Z = –1.68	0.093
Lymphocytes	1.52 (1.11–2.00)	1.68 (1.19–2.18)	Z = –1.87	0.061
Monocytes	0.62 (0.48–0.80)	0.59 (0.45–0.76)	Z = –1.35	0.178
RDW (%)	13.4 (12.9–14.2)	13.3 (12.8–14.0)	Z = –1.25	0.212
MPV (fL)	10.2 (9.5–11.0)	10.1 (9.4–10.9)	Z = –0.87	0.387
CRP (mg/L)	8.6 (2.4–27.6)	16.2 (5.1–60.4)	Z = –4.75	< 0.001
NLR	4.33 (2.66–7.68)	3.29 (2.21–6.12)	Z = –4.71	< 0.001
PLR	143.5 (99.1–210.3)	130.1 (93.2–193.7)	Z = –3.70	0.00024
LMR	2.26 (1.56–3.47)	2.36 (1.59–3.36)	Z = –0.97	0.331
SII (×10³)	995.7 (581.5–1669.0)	687.3 (463.0–1407.8)	Z = –4.65	< 0.001
SIRI	2.89 (1.63–5.26)	2.32 (1.42–5.31)	Z = –3.04	0.0025
PIV	704.7 (376.0–1258.5)	546.7 (292.2–1172.0)	Z = –2.60	0.0093
TyG–BMI	282.5 (256.3–309.1)	279.7 (254.8–299.6)	Z = –3.93	< 0.001

**Figure 2:**
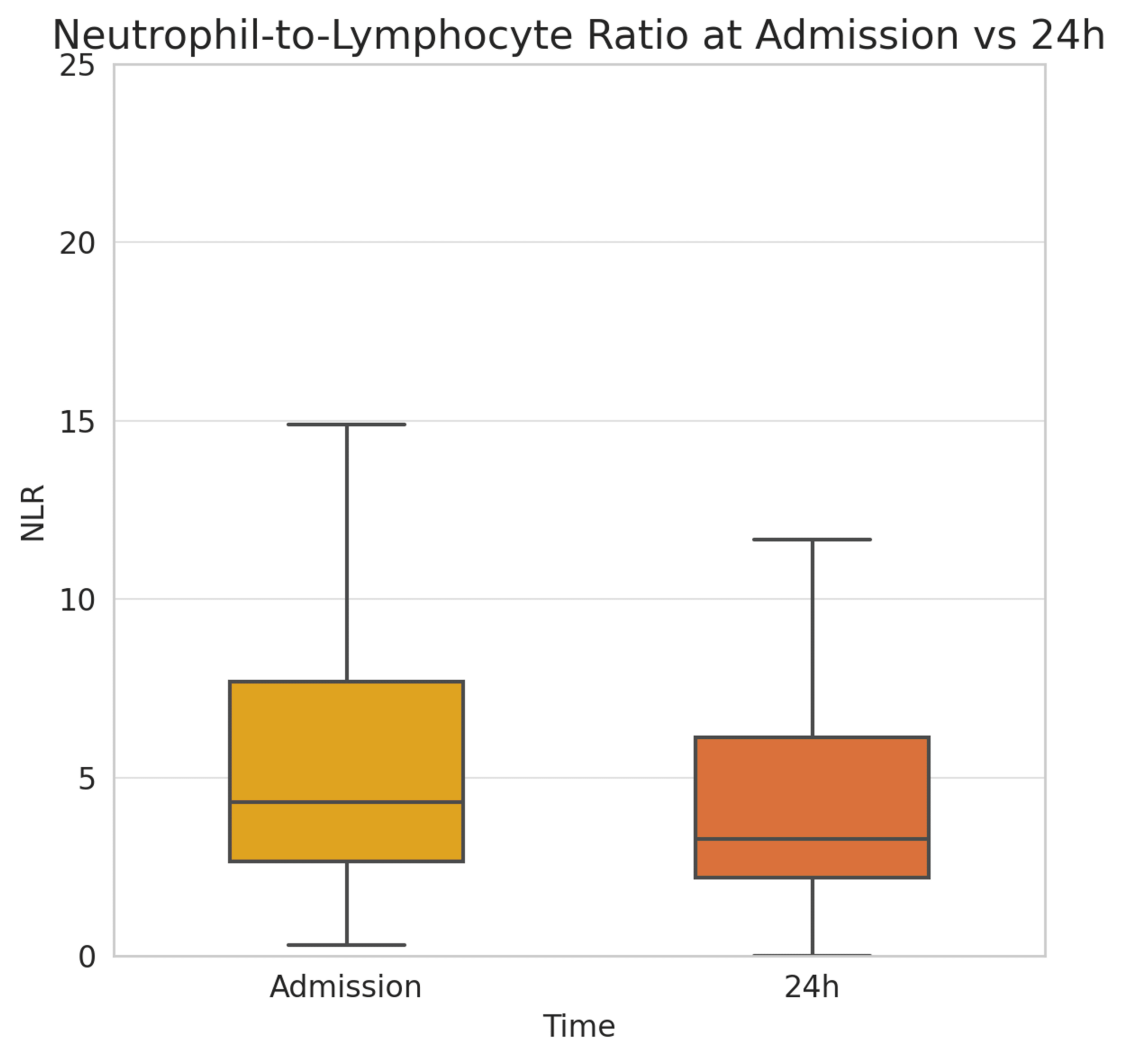
Box plot of the NLR at admission vs. 24 hours. The median NLR was lower at 24 hours compared to admission (3.3 vs. 4.3, p<0.001), and the IQR range shifted downward, indicating a general reduction in systemic inflammation after initial treatment. NLR, neutrophil-to-lymphocyte ratio; IQR: interquartile range.

A comparison between patients with and without MACE revealed no significant differences in baseline inflammatory or metabolic indices. The average admission NLR was 4.38 for patients with MACE and 4.28 for those without (p = 0.67), and similar patterns were seen for PLR, SII, SIRI, PIV, LMR, CRP, and TyG-BMI (all p > 0.3). However, within 24 hours, emerging trends were noted. Patients who later developed MACE exhibited persistently higher inflammatory indices, although the differences did not reach statistical significance. The median 24-hour NLR was 3.80 vs. 3.09 (p = 0.09), SII was 873 × 10³ vs. 641 × 10³ (p = 0.08), and CRP was 30.8 vs. 13.1 mg/L (p = 0.12), respectively (Figure 2). As shown in Table 2, patients who developed MACE had smaller declines in SIRI (ΔSIRI: 0.00 vs. -0.64, p = 0.002) and PIV (-12 vs. -64, p < 0.05) over 24 hours compared to those without MACE. TyG-BMI also remained higher in the MACE group at both time points. These trends support the hypothesis that insufficient resolution of inflammation and metabolic stress is associated with adverse outcomes.

Notably, patients without MACE demonstrated a greater reduction in specific indices during the first 24 hours. The change in SIRI (ΔSIRI) was -0.64 in the no-MACE group compared to approximately 0.00 in the MACE group (p = 0.002), and the decrease in PIV (ΔPIV) was -64 vs. -12, respectively (p < 0.05). Similar trends were observed for ΔNLR (-1.26 vs. -0.58, p = 0.04) and ΔSII (-354 vs. -149 ×10³, p = 0.03), while ΔCRP showed a non-significant difference (p = 0.07). These findings suggest that insufficient resolution of neutrophil- and monocyte-driven inflammation may be associated with adverse short-term outcomes.

**Figure 3:**
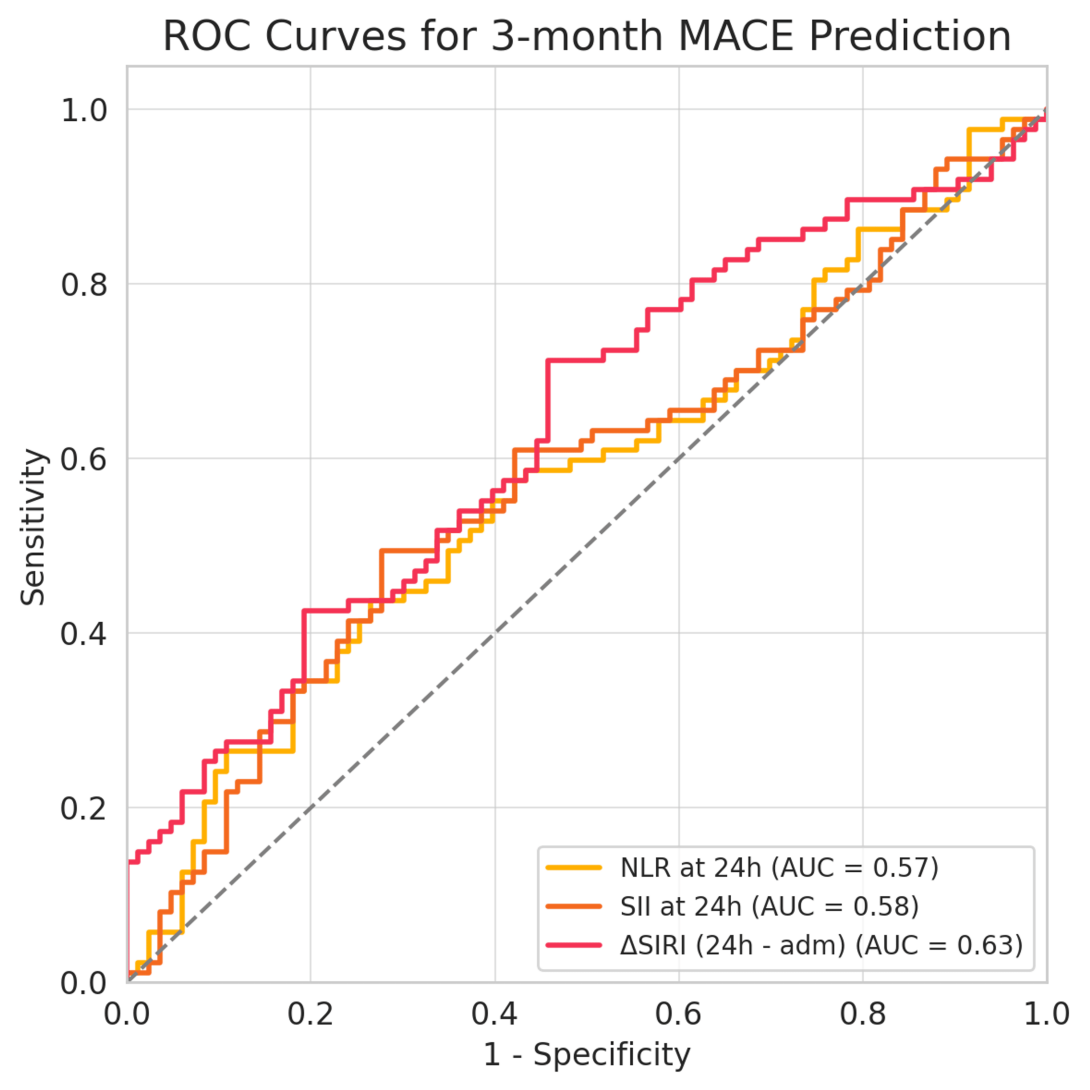
ROC curves for the prediction of three-month MACE based on selected inflammatory indices. The red curve represents the change in SIRI from admission to 24 hours (ΔSIRI) as a predictor (AUC = 0.63), the orange curve is SII at 24 hours (AUC = 0.58), and the yellow curve is NLR at 24 hours (AUC = 0.57). All three show poor to fair discrimination, as their AUCs are only slightly above 0.50. The diagonal gray line denotes the line of no discrimination (AUC = 0.50). ROC, receiver operating characteristic; MACE, major adverse cardiovascular events; NLR, neutrophil-to-lymphocyte ratio; SIRI, systemic inflammation response index; SII, systemic immune-inflammation index; AUC: area under the curve.

Receiver operating characteristic (ROC) analysis further supported these observations. While baseline values of inflammatory markers had limited discriminative power (AUCs between 0.49 and 0.55), the 24-hour values showed a slight improvement (NLR AUC = 0.57; SII AUC = 0.58). ΔSIRI demonstrated the highest AUC value among tested indices, though overall discrimination remained modest (AUC = 0.63, 95% CI: 0.54-0.71), followed by SII at 24 hours (AUC = 0.58, 95% CI: 0.49-0.67) and NLR at 24 hours (AUC = 0.57, 95% CI: 0.48-0.66) (Figure 3). Although still within a modest range, these results suggest that dynamic changes in inflammatory indices over time, rather than static absolute values, may offer better prognostic value in NSTEMI.

## Discussion

This prospective cohort study illustrates the prognostic value of early changes in inflammatory and metabolic indices among patients with NSTEMI. The findings demonstrate that patients who subsequently developed MACE exhibited distinct patterns of biomarker evolution during the first 24 hours of hospitalization. The observed 51.2% MACE rate likely reflects the inclusion of high-risk, unselected patients admitted to a tertiary referral center, many of whom experienced delays in definitive revascularization. Inflammatory indices such as NLR, PLR, PIV, and SIRI showed directional temporal trends suggestive of divergence between groups, although not all between-group differences reached statistical significance at 24 hours. Additionally, TyG-BMI demonstrated a modest association with outcomes and limited discriminatory capacity; however, its continued presence in the MACE group indicates potential relevance.

Systemic inflammation plays a pivotal role in the pathogenesis of plaque destabilization, myocardial necrosis, and subsequent ventricular remodeling following ACS events [14]. Biomarkers such as NLR and PLR, which are easily obtained from routine complete blood counts, serve as inexpensive and reproducible indicators of immune activation [15]. In our study, NLR values decreased more prominently in patients who did not develop MACE, while remaining elevated in those with adverse outcomes, suggesting a persistently heightened inflammatory response [16].

Composite indices such as PIV and SIRI, which integrate neutrophils, lymphocytes, monocytes, and platelets, provide a broader perspective on both innate and adaptive immune responses [17]. Monocytes, as part of both SIRI and PIV, play a central role in atherogenesis and post-infarction inflammation by promoting plaque instability, endothelial dysfunction, and adverse ventricular remodeling. The sustained elevation of SIRI among patients who experienced MACE is consistent with previous reports linking this marker to adverse prognosis and progression toward heart failure [18]. Similarly, elevated PIV values at 24 hours were associated with an increased likelihood of MACE, supporting its utility as a prognostic indicator in acute ischemic conditions [19].

The LMR and PLR demonstrated divergent trajectories. A lower LMR and a consistently high PLR in the MACE group may indicate a state of increased inflammation and blood clotting, even though the change in PLR was not significantly different between the groups [20].

Metabolic dysfunction is increasingly recognized as a critical driver of poor outcomes in ACS. TyG-BMI, a marker that integrates insulin resistance and anthropometric burden, has been associated with subclinical atherosclerosis and adverse cardiovascular events [21]. In our cohort, patients who developed MACE had consistently higher TyG-BMI values both at admission and after 24 hours. Although the absolute change was modest, it is physiologically plausible in the acute setting, and the persistent elevation underscores the role of early metabolic stress in early post-infarction risk [22].

Importantly, our findings suggest that temporal changes in inflammatory indices, rather than their baseline levels, may offer greater prognostic utility. Specifically, patients who remained free of events demonstrated a more pronounced reduction in SIRI and PIV within the first 24 hours. This observation aligns with studies demonstrating that the resolution of inflammation during the early phase of ACS is associated with improved myocardial healing and fewer complications [23,24]. Sustained elevation in SIRI likely reflects persistent neutrophil and monocyte activation, which can drive adverse ventricular remodeling, while elevated TyG-BMI implies continued insulin resistance and metabolic stress, both of which contribute to poor recovery.

Wang et al. reported that serial NLR measurements following PCI provided superior prognostic information compared to a single baseline value [24]. Similarly, Jenča et al. found that a declining trend in SIRI was associated with reduced incidence of heart failure and arrhythmias after NSTEMI [25]. These data reinforce the concept that serial trends in inflammatory and metabolic markers may enhance early risk stratification and therapeutic decision-making, particularly in patients with NSTEMI. However, their prognostic value in other ACS settings, such as STEMI or post-PCI cohorts, remains to be validated [26-28].

Limitations

This study has several limitations. It was conducted as a single-center, prospective cohort, which may limit the external validity and generalizability of the findings to broader and more heterogeneous populations. The relatively short follow-up period of three months captures early cardiovascular outcomes but does not provide information on long-term events or disease progression. Although the study assessed a broad panel of hematologic and metabolic indices, it did not include direct measurements of key pro-inflammatory cytokines, such as interleukin-6 (IL-6) or tumor necrosis factor-alpha (TNF-α), nor cardiac-specific biomarkers, such as high-sensitivity troponin I (hs-TnI), primarily due to logistical and cost-related constraints. The absence of such markers may limit deeper mechanistic interpretation.

Furthermore, treatment modalities were not standardized. The use of antiplatelet agents, statins, and revascularization strategies was based on physician discretion and was not controlled or stratified in the analysis. This heterogeneity may have influenced both biomarker dynamics and clinical outcomes. Additionally, no data transformations or sensitivity analyses were applied, and multivariable adjustment for revascularization or comorbidities was not performed due to sample size constraints.

## Conclusions

This study suggests that early changes in selected hematologic and metabolic-inflammatory indices, especially the reduction in SIRI and PIV during the first 24 hours, may have modest prognostic value for cardiovascular outcomes in NSTEMI patients. Although TyG-BMI remained consistently higher among patients who developed MACE, its predictive contribution was limited. Other markers, such as NLR, PLR, and SII, showed directional trends; however, between-group differences were not statistically significant in all comparisons. These findings reflect the potential role of dynamic inflammatory and metabolic changes in early risk assessment following myocardial infarction. Future studies should focus on serial biomarker profiling, adjusting for treatment strategies, to better define causality and guide clinical decision-making.

## References

[1] Basit H, Malik A, Huecker MR (2023). Non-ST-segment elevation myocardial infarction. StatPearls [Internet].

[2] Rymer JA, Tempelhof MW, Clare RM (2018). Discharge timing and outcomes after uncomplicated non-ST-segment elevation acute myocardial infarction. Am Heart J.

[3] Mănescu IB, Pál K, Lupu S, Dobreanu M (2022). Conventional biomarkers for predicting clinical outcomes in patients with heart disease. Life (Basel).

[4] Wang H, Liu Z, Shao J (2020). Immune and inflammation in acute coronary syndrome: molecular mechanisms and therapeutic implications. J Immunol Res.

[5] Henein MY, Vancheri S, Longo G, Vancheri F (2022). The role of inflammation in cardiovascular disease. Int J Mol Sci.

[6] Bai YY, Xi Y, Yin BB, Zhang JH, Chen F, Zhu B (2023). Reference intervals of systemic immune-inflammation index, neutrophil-to-lymphocyte ratio, lymphocyte-to-monocyte ratio, and platelet-to-lymphocyte ratio during normal pregnancy in China. Eur Rev Med Pharmacol Sci.

[7] Held C, Hadziosmanovic N, Aylward PE (2022). Body mass index and association with cardiovascular outcomes in patients with stable coronary heart disease - a STABILITY substudy. J Am Heart Assoc.

[8] Zhao Q, Zhang TY, Cheng YJ, Ma Y, Xu YK, Yang JQ, Zhou YJ (2021). Triglyceride-glucose index as a surrogate marker of insulin resistance for predicting cardiovascular outcomes in nondiabetic patients with non-ST-segment elevation acute coronary syndrome undergoing percutaneous coronary intervention. J Atheroscler Thromb.

[9] Bilgin M, Akkaya E, Dokuyucu R (2024). Evaluation of inflammatory markers in predicting coronary complexity: insights from the SYNTAX II score in NSTEMI patients. J Clin Med.

[10] Ghossein MA, de Kok JW, Eerenberg F (2024). Monitoring of myocardial injury by serial measurements of QRS area and T area: the MaastrICCht cohort. Ann Noninvasive Electrocardiol.

[11] Gosav EM, Tanase DM, Buliga-Finis ON, Rezuș II, Morariu PC, Floria M, Rezus C (2024). The prognostic role of the neutrophil-to-lymphocytes ratio in the most frequent cardiovascular diseases: an update. Life (Basel).

[12] Pál K, Mănescu IB, Lupu S, Dobreanu M (2023). Emerging biomarkers for predicting clinical outcomes in patients with heart disease. Life (Basel).

[13] Mulvihill NT, Foley JB (2002). Inflammation in acute coronary syndromes. Heart.

[14] Lei Y, Cao C, Tang R, Liu Y (2025). Peripheral blood inflammatory biomarkers neutrophil/lymphocyte ratio, platelet/lymphocyte ratio and systemic immune-inflammation index/albumin ratio predict prognosis and efficacy in non-small cell lung cancer patients receiving immunotherapy and opioids. BMC Cancer.

[15] Zhang Y, Yue Y, Sun Z (2025). Pan-immune-inflammation value and its association with all-cause and cause-specific mortality in the general population: a nationwide cohort study. Front Endocrinol (Lausanne).

[16] Qu C, Li X, Gao H (2023). The impact of systemic inflammation response index on the prognosis of patients with ST-segment elevation myocardial infarction undergoing percutaneous coronary intervention. Rev Cardiovasc Med.

[17] Ozilhan MO, Çakmak Karaaslan O, Acikgoz SK, Selcuk H, Selcuk MT, Maden O (2023). Systemic inflammation response index is associated MACE in patients with NSTEMI. Eur Rev Med Pharmacol Sci.

[18] Kumar R, Shah JA, Solangi BA (2022). The burden of short-term major adverse cardiac events and its determinants after emergency percutaneous coronary revascularization: a prospective follow-up study. J Saudi Heart Assoc.

[19] Cai M, Liang D, Gao F (2020). Association of lymphocyte-to-monocyte ratio with the long-term outcome after hospital discharge in patients with ST-elevation myocardial infarction: a retrospective cohort study. Coron Artery Dis.

[20] Bessho R, Kashiwagi K, Ikura A, Yamataka K, Inaishi J, Takaishi H, Kanai T (2022). A significant risk of metabolic dysfunction-associated fatty liver disease plus diabetes on subclinical atherosclerosis. PLoS One.

[21] Platt C, Houstis N, Rosenzweig A (2015). Using exercise to measure and modify cardiac function. Cell Metab.

[22] Libby P, Tabas I, Fredman G, Fisher EA (2014). Inflammation and its resolution as determinants of acute coronary syndromes. Circ Res.

[23] Nardin M, Verdoia M, Laera N, Cao D, De Luca G (2023). New insights into pathophysiology and new risk factors for ACS. J Clin Med.

[24] Wang J, Hu S, Liang C, Ling Y (2022). The association between systemic inflammatory response index and new-onset atrial fibrillation in patients with ST-elevated myocardial infarction treated with percutaneous coronary intervention. BMC Cardiovasc Disord.

[25] Jenča D, Melenovský V, Stehlik J (2021). Heart failure after myocardial infarction: incidence and predictors. ESC Heart Fail.

[26] Liu H, Wang J, Wang W, Ruan M, Liu J (2025). Association between metabolic and inflammatory biomarkers and prognosis in traumatic brain injury: a focus on short- and medium-term mortality. J Inflamm Res.

[27] Netala VR, Hou T, Wang Y, Zhang Z, Teertam SK (2025). Cardiovascular biomarkers: tools for precision diagnosis and prognosis. Int J Mol Sci.

[28] Matter MA, Paneni F, Libby P (2024). Inflammation in acute myocardial infarction: the good, the bad and the ugly. Eur Heart J.

